# Reimagining oral health professionalism in Africa: a narrative from the margins

**DOI:** 10.3389/fmed.2026.1834418

**Published:** 2026-05-22

**Authors:** Moréniké Oluwátóyìn Foláyan, Opeyemi Oyewunmi Ekundayo, Nceba Gqaleni

**Affiliations:** 1 Department of Child Dental Health, Obafemi Awolowo University, Ilé-Ifẹ̀, Nigeria; 2 Department of Public Health Dentistry, Saveetha Dental College and Hospitals, Saveetha Institute of Medical and Technical Sciences, Saveetha University, Chennai, India; 3 Centre for Gender and Social Policy Studies, Obafemi Awolowo University, Ilé-Ifẹ̀, Nigeria; 4 Sub-Discipline of Traditional Medicine, University of KwaZulu-Natal, Durban, South Africa; 5 Africa Health Research Institute, Durban, South Africa

**Keywords:** decolonisation, epistemic injustice, feminist epistemology, indigenous knowledge systems, oral health professionalism, relational pedagogy, Ubuntu philosophy, Yorùbá culture

## Abstract

**Background:**

The Western Enlightenment model of oral health professionalism in Africa, emphasizing clinical detachment and biomedical reductionism, fails to resonate with communities like the Yorùbá, where oral health is intertwined with spiritual personhood and relational knowing. This paper critiques colonial epistemological foundations underpinning oral health professionalism, articulates an Ubuntu-informed alternative grounded in Yorùbá knowledge, and proposes a relational framework centering marginalized community.

**Methods:**

We conducted a critical interpretive synthesis to integrate insights from community-based participatory research in Yorùbá communities, feminist epistemology, African philosophy, and health professions education through iterative dialogue between empirical findings and theoretical frameworks. We examined colonial epistemologies and articulated a relational alternative for oral health professionalism.

**Results:**

Oral health professionalism in Africa remains shaped by colonial epistemologies that privilege clinical detachment while excluding indigenous knowledge systems. An Ubuntu-informed alternative, attentive to feminist critiques of gendered power relations, offers a foundation through mutual vulnerability, clinical empathy, and ecological professional identity formation across interdependent domains. Transformations across curriculum, assessment, workforce policy, research, and governance are required to address epistemic injustices.

**Conclusion:**

Reimagining oral health professionalism in Africa requires approaches holding clinical excellence and relational depth, recognizing professional identity as formed through interdependent relationships. Dental education must transform by reconceptualising itself as an ecology of relations cultivating practitioners accountable to communities as both knowers and learners.

## Introduction

The question of what it means to be a professional in oral health appears deceptively simple, yet its answer carries the accumulated weight of centuries. In Africa, dental students learn to embody professionalism through clinical detachment, universal ethical principles, and individually accountable practice (1, 2), using standards that bear the invisible watermark of their origins in Western Enlightenment thought, colonial health system architecture, and epistemologies that privilege cognitive rationality over relational ways of knowing that are often dismissed as myths (3, 4). These frameworks present themselves as value-neutral while carrying deeply specific assumptions about knowledge, authority, and the proper relationship between healer and the healed.

However, when one listens instead to the oral health narratives circulating among communities, like the Yorùbá communities in Southwest Nigeria, a different truth begins to emerge. The Yorùbá worldview centers on a holistic concept of personhood comprising *ara* (the body), *ẹ̀mí* (the life force), and *orí* (the inner head or spiritual essence), wherein the mouth’s care becomes integral to nurturing the *ẹ̀mí* and harmonizing the *orí* with the physical self (5, 6). Traditional songs reinforce this connection daily, linking oral hygiene with broader values of dignity, self-care, and moderation (6). Divination poems such as *Òdí Méjì* emphasize that dazzling white teeth are synonymous with good health, a physical manifestation of spiritual balance (6). Yet despite this cultural reverence for oral health, modern dental service utilization in Southwest Nigeria remains low (6). This paradox between cultural value and practical access raises a question: whose knowledge counts as professional, and more urgently, how might we reimagine oral health professionalism through the embodied experiences of those most marginalized by current systems?

The answer to this question becomes clearer when examining the structural and educational landscape that shapes oral health provision. The health landscape in Africa is a colonial residue, visible in the urban-centric maldistribution of infrastructure, the privileging of curative over preventive approaches, and the epistemic hierarchy that marginalizes health within primary care (7–9). Dental services were established to serve the needs of colonial administrators and urban elites, with minimal investment in the health infrastructure of rural or indigenous populations (4). Professional identity formation in this context becomes a process of assimilation into imported models, where students learn to value technical proficiency above all else, to maintain emotional detachment as a marker of professionalism (10), and to view patients through biomedical lenses that fragment bodies into discrete clinical problems (11). It is this fragmentation that creates such disjuncture within communities such as the Yorùbá communities: the mouth that clinicians treat as a pathological entity is, for patients, a spiritual entity whose care remains inseparable from broader concepts of personhood and social identity (5, 6). This disjuncture between professional training and community worldviews represents a cultural gap and epistemic violence through the systematic delegitimization of ways of knowing that fall outside Western biomedical frameworks. Addressing this violence requires an alternative foundation for professionalism.

This paper aims to: (1) critique the Western epistemological foundations underpinning oral health professionalism in Africa; (2) articulate an Ubuntu-informed alternative grounded in Yorùbá cultural knowledge; and (3) propose a relational, ecologically-oriented framework for oral health professional formation that centers the experiences and knowledges of marginalized communities.

## Methodology

The preceding section described the structural residues of colonial health systems. However, describing structure is insufficient; we require analytic tools to understand how knowledge itself is delegitimized. We employed a critical interpretive synthesis approach, drawing on: (a) the authors’ collective decades of community-based participatory research in Yorùbá communities; (b) systematic engagement with philosophical and theoretical literatures across feminist epistemology, African philosophy, and health professions education; and (c) iterative dialogue between empirical findings from our research program and theoretical frameworks. The narrative presented emerges from knowledge produced through sustained engagement with marginalized perspectives (12).

This critical interpretive synthesis followed an iterative process. First, as we identified two focal questions: (1) How do colonial epistemologies shape oral health professionalism in Africa? (2) What alternative frameworks emerge from Yorùbá and Ubuntu knowledge systems? Next, we identified related constructs: clinical detachment, relational ontology, epistemic hierarchy, gendered knowledge, and community accountability, and then mapped these constructs feminist epistemological critiques to reveal how the disembodied knower ideal systematically excludes indigenous knowledge. Third, we articulated Ubuntu as a relational foundation for reimagined professionalism, integrating feminist cautions against romanticizing community. Fourth, we demonstrated how epistemic justice requires centering marginalized knowledge, using jédijédi as an illustrative case. Fifth, we translated these theoretical insights into concrete transformations across curriculum, assessment, workforce, research, and governance. Finally, we acknowledge limitations and propose future research agendas. Each section builds on the previous: the colonial legacy creates the problem, Ubuntu offers an alternative, feminist critiques refine that alternative, epistemic justice specifies the harm, and practical implications to operationalize the solution.

Our approach is necessarily interdisciplinary and reflexive, acknowledging that the authors’ own positionalities, as oral health researchers, social scientists, and traditional medicine scholars based in African institutions, shape the interpretations offered. Rather than claiming value-neutrality, we embrace situated knowledge production as a strength, recognizing that insights from the margins require sustained engagement with the communities whose knowledge has been systematically delegitimized. We three scholars from Nigeria and South Africa, with diverse disciplinary training (dentistry, gender studies, traditional medicine) and deep community engagement, collectively shaped the paper’s critique of Western professionalism. Folayan (Yorùbá female dentist) highlighted insider-outsider epistemic tensions; Ekundayo (Yorùbá gender scholar) ensured feminist critique of patriarchal traditions in Ubuntu; Gqaleni (South African Ubuntu scholar) provided discuss on relational ontology and clinical empathy as alternatives to detached concern.

## Feminist epistemology and the disembodied knower

To begin this sequential analysis, we first turn to the analytic tools that expose how colonial epistemologies operate within oral health professionalism. Feminist epistemology provides such tools, particularly the concept of the disembodied knower (13, 14), the ideal that genuine knowledge requires abstraction from identity, experience, and social location. Sandra Harding’s critique of traditional objectivity illuminates what is at stake: the assumption that proper method can neutralize cultural biases ignores how social location shapes what we can notice (3, 15). Professionals working within dominant frameworks may therefore remain systematically blind to phenomena visible only from marginalized perspectives.

This systematic blindness is not merely theoretical; research across communities in Africa reveals the depth of cultural knowledge systematically excluded from professional training. One example is the poor integration of indigenous knowledge about the use of plants for oral health. Ethnobotanical surveys have documented numerous plant species useful for oral healthcare in Africa, including remedies derived from roots, leaves, stems, barks, and fruits administered through topical application, mouth rinses, gargling, chewing, and inhalation (16). The Solanaceae family, rich in biologically active alkaloids with antimicrobial properties validated against cariogenic organisms, including *Streptococcus mutans*, features prominently in these traditional practices (17). Yet this knowledge circulates outside formal health systems, is delegitimized within professional training, and is positioned as a belief to be corrected rather than as a resource for culturally grounded care.

What this illustrates is how the disembodied knower ideal produces systematic epistemic blindness: professionals trained to value abstract, universalizable knowledge cannot recognize the situated, embodied expertise that communities possess. This blindness has material consequences for oral health outcomes, as patients whose knowledge systems are dismissed may delay seeking care, adhere poorly to treatment recommendations, and remain alienated from health systems designed without their worldviews in mind.

## Ubuntu as a relational foundation

African philosophical traditions offer rich resources for reimagining professionalism from the ground up. Ubuntu, often translated as “I am because we are,” articulates a relational ontology fundamentally different from the individualism undergirding Western professional codes (18, 19). Within Ubuntu, personhood is constituted through relationships with others; one’s humanity remains entangled with those around one. As Folayan and Haire (20) demonstrated in the context of the African communitarian society, this relational ontology has direct implications for public engagement, where ethical practice requires moving beyond individual autonomy toward collective accountability and participatory decision-making. For oral health, a professionalism grounded in Ubuntu would begin with the recognition of mutual vulnerability and interdependence, understanding that the dental professional’s own wellbeing is bound up with their patients’ wellbeing, and that healing occurs in relationship rather than through technical intervention alone.

This philosophy de-emphasizes clinical detachment as the marker of professionalism and captures a fundamental reorientation in how professional identity might be understood within oral health education and practice. Clinical detachment, historically positioned as essential to objective clinical judgment and professional boundary maintenance (21), has been increasingly critiqued for creating relational distances that can undermine rather than support therapeutic effectiveness (22). Within dental education specifically, the emphasis on emotional distancing has been shown to correlate with reduced patient trust, diminished treatment adherence, and lower satisfaction among patients from culturally diverse backgrounds (23, 24).

Ubuntu’s relational ontology offers an alternative foundation. If personhood is constituted through relationships, then the clinical encounter is a space of mutual becoming where both practitioner and patient are transformed rather than a meeting between autonomous individuals (25). This aligns with emerging evidence that patients’ health outcomes are influenced by the quality of the practitioner-patient relationship, including perceived empathy, trust, and collaborative decision-making (26, 27). When professionalism is reframed through Ubuntu, the practitioner’s capacity for clinical empathy and the willingness to be emotionally engaged while maintaining reflective awareness matters (22).

This relational understanding resonates with recent calls in health professions education to move beyond competency-based frameworks toward approaches that center relational virtues such as attentiveness, responsiveness, and mutual accountability (28, 29). In the South African context, the Truth and Reconciliation Commission’s emphasis on Ubuntu as a national ethic prompts the reconsideration of how health professionals might be formed differently: as witnesses to suffering rather than detached technicians, as participants in community healing rather than individual treatment providers (30, 31).

For dental education specifically, Ubuntu-informed professionalism would require curriculum reform that creates opportunities for students to develop relational capacities through sustained engagement with communities, critical reflection on power dynamics in clinical encounters, and explicit attention to how professional identity is shaped by cultural context. It would also necessitate assessment practices that value relational competence alongside technical proficiency, recognizing that the ability to be present with patients in their vulnerability is a core professional capability rather than a soft skill (32). The challenge is that such reforms require confronting the hidden curriculum that continues to rewards detachment and positions relational engagement as somehow less rigorous than technical intervention (33).

The question remains whether oral health professions education can rise to this challenge. Can we create curricula that prepares oral health professionals not only to treat disease but to understand how disease is produced by social structures? Can we train practitioners who see themselves as accountable to communities as well as to professional bodies? Can we center epistemologies that have been marginalized, voices that have been silenced, experiences that have been dismissed as anecdotal rather than evidentiary? This requires more than adding cultural competence modules to existing curricula; it requires reorientation of how we understand professional identity formation. It requires acknowledging that the professional is both a knower and a learner, a healer and a partner in healing, an individual and a participant in communities of care. It requires reconceptualizing dental education itself as an ecology of relations, wherein pedagogical possibility and professional flourishing emerge through the interdependence of multiple relational domains, and wherein the conditions that make such ecological relations possible are deliberately cultivated, sustained, and defended against the narrowing logics of market accountability and epistemic hierarchy.

This envisioned curriculum resonates with recent calls in educational scholarship to reconceptualize pedagogy as an ecology of relations, wherein learning and professional formation are understood as emerging through interdependent relational domains rather than through isolated educator-learner dyads (34). Just as Liang et al. argue that early childhood education must attend to children’s self-relations, peer relations, family relations, and more-than-human relations as constitutive of pedagogical possibility, so too must dental education recognize that professional identity is formed through both clinical instruction and through the entire relational ecology in which students learn: their relationships with themselves as knowers, with patients and communities, with peers and mentors, and with the broader cultural and material worlds they inhabit. This vision also resonates with the growing recognition that authentic community engagement in Africa requires understanding its philosophical foundations (35). Ubuntu thus offers a relational ontology that directly counters the disembodied knower ideal.

## Integrating feminist critiques within Ubuntu

However, this is not to romanticize Ubuntu or suggest its uncritical application. African feminist scholars have rightly cautioned against idealizing tradition in ways that obscure internal hierarchies and exclusions (36, 37). Communities are not uniformly benign spaces of mutual recognition but are also sites of gendered power relations. Studies among Yorùbá communities reveal how gender shapes oral health experiences: women’s health concerns may be normalized as maternal sacrifice, their mobility restricted, and their pain dismissed (6). The cultural construct of *jédijédi*, an ethnomedical phenomenon relevant to dental caries risk, is predominantly framed as a male-centered affliction linked to sexual dysfunction, yet women challenge this narrative by highlighting menstrual irregularities, infertility, and childbirth complications as consequences of the condition (38).

These specific gendered dynamics within Yorùbá communities are central warnings for any professionalism claiming an Ubuntu foundation. If Ubuntu-informed oral health professionalism is to avoid replicating the very hierarchies it seeks to dismantle, it must be accountable to feminist critiques that reveal how the community often demands disproportionate sacrifice from women and girls, making the community a site of gendered inequality rather than liberation (36). Colonial and postcolonial constructions of gender have overwritten more fluid indigenous understandings, creating hierarchies that position women’s bodies and labor as resources for communal welfare rather than recognizing their full personhood (37).

For the practitioner committed to Ubuntu-informed professionalism, these insights demand recognizing that cultural sensitivity cannot mean uncritical acceptance of practices that harm women. Clinical encounters must actively create space for women’s voices and experiential knowledge, treating them as authoritative interpreters of their own bodies rather than passive recipients of professional expertise. It requires practitioners to examine how their own positionality may reproduce hierarchies, aligning with the ecological understanding that shifts or ruptures in one relational domain, such as gendered power dynamics within families, reverberating across others, shaping what becomes possible in clinical encounters and professional formation alike (34). Feminist interventions thus prevent Ubuntu from being deployed to justify harm.

## Epistemic justice and marginalized knowledges

Change requires that the hidden curriculum of health professions education be recognized, as it powerfully shapes who counts as a knower. Students learn from formal instruction, from observation of clinical teachers, from institutional routines, and from whose knowledge is celebrated and dismissed. Miranda Fricker conceptualizes this as epistemic injustice: the wrong done to someone specifically in their capacity as a knower (39). This could be in the form of testimonial injustice when prejudice causes a hearer to give a speaker’s words less credibility; or as hermeneutical injustice when gaps in collective interpretive resources disadvantage sense-making.

In Yorùbá communities, sophisticated oral health knowledge circulates outside formal systems: mothers learn from elders about managing teething symptoms with herbal preparations (40, 41); traditional birth attendants possess knowledge about natal teeth (42); community members use chewing sticks with documented antimicrobial properties, and saltwater rinses, and other remedies validated by generations of use (43). Yet this knowledge is systematically delegitimized within professional training, positioned as a misconception to be corrected rather than as a resource for culturally grounded care.

Dotson’s concept of contributory injustice specifies what is at stake: when hermeneutical resources developed by marginalized groups are systematically ignored, the result is active harm (44, 45). The dismissal of grandmothers’ oral health wisdom as superstition leads to the loss of that knowledge from clinical practice and it erodes confidence in knowledge traditions that have sustained communities for generations. A professionalism capable of addressing this harm would require the practitioner to recognize that they have as much to learn from communities as to teach them. This is what Harding calls strong objectivity: the recognition that knowledge is strengthened, not weakened, by acknowledging its situatedness and incorporating multiple perspectives, especially those from marginalized locations (3, 46). In ecological terms, this means recognizing that the relational domain of patient-community relations is a constitutive site where pedagogical and professional meanings are made and remade (34).

The practical implications are illustrated by research on cultural sensitivity during fieldwork in Ile-Ife. Investigators conducting oral health surveys among Yorùbá mother-child pairs encountered cultural norms that infringed on participants’ privacy and created mistrust, including caregivers’ concerns about dentists touching children’s heads during height measurement (47). These challenges reveal the gap between professional protocols designed in abstraction from cultural context and the lived realities of practice in specific communities. Professionalism informed by strong objectivity would anticipate such challenges through community mapping, engagement with local leaders, and methodological adaptations that respect cultural norms while maintaining scientific rigor.

Participatory methods offer pathways to center marginalized voices. Among Yorùbá communities, studies have documented cultural themes through diverse approaches: ethnographic investigations of neonatal care (48), community-based participatory exploration of *jédijédi* beliefs (38), and systematic scoping reviews of cultural determinants (16). The Community of Practice approach enabled collaborative knowledge generation (38), embodying the principle that knowledge is constructed through relational exchanges and mirroring the relational ethic essential for equitable research collaborations. This approach recognizes what Liang et al. (34) term peer relations as sites of collective meaning-making, challenging adult-centric assumptions about where knowledge and belonging are formed. In dental education, this suggests that students’ professional learning should be co-constructed through hierarchical relationships with clinical teachers, horizontal relationships with peers, shared negotiation of clinical challenges, and collaborative sense-making that draws on diverse cultural and experiential resources.

The cultural construct of *jédijédi* exemplifies the epistemic resources available from community knowledge. This ethnomedical condition, understood within Yorùbá communities as a syndrome affecting multiple body systems and associated with sugar consumption, shapes household behaviors in ways that may inadvertently reduce children’s exposure to cariogenic foods (38). Parents restrict sugary snacks and administer bitter herbal preparations after children consume sweets. The knowledge embodied in these practices about diet and health, about the protective effects of bitter herbs, and about intergenerational transmission of norms, represents a resource that professionalism committed to equity would seek to understand and engage.

Among Yorùbá communities, initiatives to develop culturally appropriate oral health education tools demonstrate what reimagined professionalism might look like. Researchers have collaborated with communities to develop oral hygiene education songs in local dialects, health educational videos addressing myths, photo-posters with captions translated into local languages, and traditional folktales presented with comic strips (49). These tools translate Western health messages into local languages by engaging with cultural frameworks to create messages that resonate within community worldviews (5). This is professionalism as lived practice: accountable to multiple knowledge systems, responsive to community priorities, and humble about the limits of any single perspective. This way of creating knowledge gives attention to materials, spaces, and ecologies through which learning and belonging are constituted, recognizing that oral health education materials themselves are relational actors that shape how knowledge is transmitted, received, and taken up within communities.

Furthermore, oral health professionalism requires decolonizing oral health care by redistributing power and creating accountability frameworks that recognize oral health as a human right (50). A rights-based, accountability-informed decolonization framework centers the experiences of marginalized communities, holds systems accountable for redressing structural violence, and empowers communities to reclaim health sovereignty. This means integrating Indigenous oral health practices with Western care through respectful, balanced approaches that value both systems, developing referral networks between Indigenous practitioners and Western providers, creating cross-training programmers, ensuring governmental support, including frameworks recognizing and funding Indigenous practitioners, and decolonizing research methodologies through community-led approaches that align with community ways of knowing, being, and doing.

## Practical implications

Reimagining professionalism requires concrete transformations across multiple interconnected domains, moving beyond theoretical critique toward actionable change within educational institutions, health systems, and research enterprises. These transformations require a reorientation of how oral health professions conceptualize their relationship with the communities they serve.

As highlighted in Table 1, the most immediate site of transformation is curriculum design, where dental education must move beyond tokenistic inclusion of cultural competence modules toward substantive integration of indigenous knowledge systems. This requires mandatory curricula taught in genuine partnership with community knowledge holders such as traditional healers, grandmothers, and local health workers, who are positioned as co-educators rather than exotic informants. Students require structured opportunities for sustained community engagement through placements that immerse them in the relational ecologies where oral health is produced: homes, markets, religious gatherings, and traditional healing spaces. These experiences develop the capacity for attending to multiple relational domains, such as self-relations, peer relations, and community relations that constitute the foundation of Ubuntu-centered practice. Such placements differ from conventional community rotations in that they position students as learners rather than experts, emphasizing receptivity and humility over technical intervention (51).

**TABLE 1:** Practical implications for reimagining oral health professionalism in Africa.

Domain	Current challenge	Transformative approach	Key actors	Illustrative examples from literature
Curriculum design	Cultural competence taught as add-on modules; community members positioned as passive subjects	Mandatory integration of indigenous knowledge systems taught in partnership with community knowledge holders; sustained community placements emphasizing reciprocal learning	Dental schools, community knowledge holders, traditional healers, and professional bodies	Community-based participatory development of oral health education songs in local dialects (49); placements where students learn from traditional birth attendants about neonatal oral care (42)
Assessment reform	Focused on technical skills; relational competencies are invisible in assessment	Include relational competencies in assessment with portfolios documenting community engagement and community feedback mechanisms, as a formal assessment.	Regulatory bodies, dental educators, and community representatives	Portfolios capturing students’ reflective engagement with *jédijédi* beliefs and their implications for caries prevention (38); community assessment of graduates’ cultural humility
Workforce policy	Traditional practitioners are unrecognized; no cross-referral mechanisms	Create accredited pathways for traditional oral health practitioners; establish regulatory frameworks enabling collaboration; fund community-led initiatives	Health ministries, professional regulatory councils, and traditional medicine boards	Nigeria frameworks recognizing traditional healers within primary care (56); integration of chewing stick promotion alongside toothbrush education (43)
Research agendas	Communities as research subjects; metrics rewards publication over community benefit	Prioritize community-based participatory research; community partners as co-investigators; metrics valuing knowledge translation to communities	Funding bodies, research institutions, ethics committees, and community advisory boards	Community of practice approach to studying *jédijédi* (38); ethnographic investigations co-designed with Yorùbá communities (60)
Institutional transformation	Hidden curricula reinforce epistemic hierarchies; no community accountability mechanisms.	Examine and shift symbolic representations; establish community governance representation; create community feedback mechanisms on graduate performance.	Dental schools, teaching hospitals, university leadership, and community advisory boards	Institutional policies requiring community representation in curriculum committees; regular community forums for institutional accountability (35)
Cross-cutting enablers	Fragmented efforts; lack of policy coherence; unsustainable pilot projects	Develop national frameworks aligning education, policy, research, and practice; ensure sustainable financing; build evidence base through implementation research.	National governments, international partners, professional associations, and communities	Rights-based, accountability-informed decolonisation frameworks (50); ecological approaches recognizing interdependence of relational domains (34)

There is also the need for assessment reform that values relational competence alongside technical proficiency. Current assessment frameworks remain overwhelmingly focused on observable technical skills, rendering invisible the relational capacities that determine whether technical interventions actually serve patients’ needs (27). Assessment methods must evolve to capture students’ ability to demonstrate clinical empathy, collaborate with traditional knowledge holders, and respond respectfully to patients’ explanatory models (52). Portfolios documenting students’ reflective engagement with community experiences, reflective journals tracing the development of cultural humility, and community feedback mechanisms that give community members an authentic voice in evaluating graduates’ readiness to practice are approaches that can complement traditional clinical evaluations by making visible what currently remains hidden (53). The challenge is that such methods resist standardization and require institutions to trust community members as credible assessors of professional competence; a trust that itself represents a decolonial shift in epistemic authority.

Beyond educational institutions, workforce policy must create formal recognition pathways for traditional oral health practitioners, acknowledging that communities already rely on these practitioners and will continue to do so regardless of professional sanction (54). This requires moving beyond rhetorical acknowledgment of traditional medicine toward substantive integration: accredited training pathways that respect indigenous pedagogies while ensuring patient safety, regulatory frameworks that enable cross-referral between traditional and biomedical practitioners, and sustainable financing mechanisms that fund community-led oral health initiatives (55). Countries such as Nigeria (56) and Zambia (57) have explored such integration through frameworks that recognize traditional healers within primary health care teams, though oral health remains notably absent from most of these initiatives. Learning from these partial successes and failures is essential for developing models that work across diverse African contexts.

Research agendas must also simultaneously transform, with funding bodies prioritizing community-based participatory research that centers marginalized epistemologies. This means positioning community partners as co-investigators rather than research participants, ensuring that research questions emerge from community-identified priorities rather than externally imposed agendas (58). Research metrics must evolve to value knowledge translation to communities through culturally appropriate educational materials, community dialogues, and practitioner training, alongside publication in high-impact journals (59). Ethical review processes should include community representatives with genuine authority to assess cultural appropriateness and community benefit (35), recognizing that institutional review boards trained in Western research ethics frameworks may lack the cultural competence to evaluate research in African community contexts.

Underpinning all these transformations is the necessity of institutional transformation within dental schools and teaching hospitals. Institutions must examine their own hidden curricula—whose knowledge is displayed on walls, whose languages are spoken in corridors, whose voices are heard in governance—and take deliberate action to shift these symbolic and material arrangements (33). When clinic walls display only Western anatomical charts and dental equipment catalogues, while traditional chewing sticks and herbal preparations remain invisible, students learn a powerful lesson about whose knowledge counts. When institutional governance includes no community representatives with decision-making authority, students learn that accountability flows upward to professional bodies rather than outward to communities. Institutional accountability to communities requires establishing mechanisms for community feedback on graduate performance, patient experience surveys translated into local languages, and regular community meetings where institutions report on their responsiveness to community-identified priorities.

These transformations are mutually reinforcing: curriculum reform prepares graduates who can work effectively with traditional practitioners, workforce policy creates the structural conditions for such collaboration, research agendas generate the evidence base for effective integration, and institutional transformation ensures that educational environments model the relational values they seek to instill. None of these domains can succeed in isolation; together, they constitute an ecology of change that recognizes the interdependence of educational, policy, research, and institutional systems in shaping professional identity and practice. Without such a comprehensive transformation, efforts to reimagine professionalism will remain marginal innovations within fundamentally unchanged systems, leaving untouched the epistemic hierarchies and structural inequities that produce oral health disparities across African communities.

## Limitations and future directions

Several limitations warrant acknowledgment. First, while we draw extensively on Yorùbá cultural frameworks, Africa’s diversity precludes generalization; Ubuntu may manifest differently across linguistic and ethnic groups, and other philosophical traditions may offer complementary or contrasting resources for reimagining professionalism. Second, the manuscript focuses on conceptual critique rather than empirical evaluation of Ubuntu-informed interventions; future research should assess whether such approaches improve oral health outcomes, reduce inequities, and prove feasible within resource-constrained health systems. Third, the tension between respecting cultural traditions and addressing harmful practices, particularly gendered hierarchies, requires ongoing negotiation not fully resolved here. Fourth, our focus on professionalism in oral health education may limit applicability to other health professions, though the theoretical framework may be applicable beyond the oral health field. Fifth, the manuscript does not fully address economic and political constraints such as neoliberal reforms, donor conditionalities, and health system underfunding that shape possibilities for curriculum reform.

Future research should: (1) develop and validate instruments for assessing relational competencies in oral health professionals; (2) conduct comparative studies across African contexts to understand how Ubuntu manifests in different cultural settings; (3) evaluate the effectiveness of community-academic partnerships in transforming professional education; (4) document existing models of traditional-Western practitioner collaboration and their impact on oral health outcomes; and (5) explore how students from diverse backgrounds experience curricula that attempt to center marginalized epistemologies.

## Conclusion

The counter-narrative proposed here does not reject professionalism but reimagines it. Professionalism remains essential for competent, ethical care, but professionalism must be understood as historically situated and culturally contested rather than as a timeless universal. It must be practiced as lived negotiation rather than rigid adherence to imported codes. This reimagined professionalism for oral health practice in Africa holds multiple dimensions in creative tension: clinical excellence with relational depth, technical proficiency with the awareness of structural determinants, individual accountability with collective responsibility. It recognizes that the oral health professional’s identity is formed in clinics, classrooms and communities, through relationships, in responsiveness to those most vulnerable to current systems’ failures. It requires developing pedagogical and policy languages that can name, support, and resource relational work across multiple interdependent domains. This means creating curricula that attend to students’ self-relations (how they come to see themselves as knowers and healers), their relationships with patients and communities (as reciprocal and co-constitutive), their peer relations (as sites of collaborative learning and identity formation), and their more-than-human relations (with the materials, technologies, and ecological systems that shape oral health). This reimagining is not merely academic. When professionalism excludes community knowledge, patients delay seeking care, adhere poorly to treatment, and suffer preventable morbidity. When curricula erase indigenous epistemologies, graduates lack the cultural humility to serve diverse populations effectively. When policies marginalize traditional practitioners, health systems forfeit trusted human resources. The stakes are therefore clinical and ethical: reimagining professionalism is inseparable from addressing oral health inequities. The margins to which we have consigned community knowledge may yet become the center from which a more just, more effective, more humane oral health professionalism emerges.

## Data Availability

The original contributions presented in this study are included in the article/supplementary material, further inquiries can be directed to the corresponding author.
